# Health–environment efficiency of diets shows nonlinear trends over 1990–2011

**DOI:** 10.1038/s43016-024-00924-z

**Published:** 2024-02-08

**Authors:** Pan He, Zhu Liu, Giovanni Baiocchi, Dabo Guan, Yan Bai, Klaus Hubacek

**Affiliations:** 1 School of Earth and Environmental Sciences, Cardiff University, Cardiff, UK; 2 Department of Earth System Science, Tsinghua University, Beijing, China; 3 Institute for Climate and Carbon Neutrality and Department of Geography, University of Hong Kong, Pok Fu Lam, Hong Kong; 4 Department of Geographical Science, University of Maryland, College Park, MD USA; 5 The Bartlett School of Sustainable Construction, University College London, London, UK; 6 School of Public Affairs, Zhejiang University, Hangzhou, China; 7 Development Data Group, the World Bank, Washington, DC USA; 8 Integrated Research on Energy, Environment & Society (IREES) at the Energy Sustainability Research Institute Groningen (ESRIG), University of Groningen, Groningen, the Netherlands

**Keywords:** Sustainability, Risk factors

## Abstract

Understanding the impacts of diets on health and the environment, as well as their association with socio-economic development, is key to operationalize and monitor food systems shifts. Here we propose a health–environment efficiency indicator defined as a ratio of health benefits and four key food-related environmental impacts (greenhouse gas emissions, scarcity-weighted water withdrawal, acidifying and eutrophying emissions) to assess how diets have performed in supporting healthy lives in relation to environmental pollution and resource consumption across 195 countries from 1990 to 2011. We find that the health–environment efficiency of each environmental input follows a nonlinear path along the Socio-Demographic Index gradient representing different development levels. Health–environment efficiency first increases thanks to the elimination of child and maternal malnutrition through greater food supply, then decreases driven by additional environmental impacts from a shift to animal products, and finally shows a slow growth in some developed countries again as they shift towards healthier diets.

## Abstract (web summary)

The coupled impacts of diets on health and the environment must be considered when setting food policy targets and evaluating the effectiveness of specific interventions. A newly proposed health–environment efficiency indicator applied to 195 countries over two decades can aid this process, revealing important trends and drivers.

## Main

Changing global dietary patterns to improve health outcomes and reduce detrimental effects on the environment has become increasingly important in global and national policy agendas^1–3^. Poor diets expose the global population to malnutrition in all its forms causing undernutrition^4^, micronutrient deficiency^5,6^ and various diet-related non-communicable diseases^7^ (for example, overweight or obesity^4,8–10^ and various other non-communicable diseases such as cardiovascular disease and type II diabetes^7^) while contributing critically to environmental impacts driven by anthropogenic greenhouse gas (GHG) emissions^4,11^, water consumption^12,13^, land occupation^13^ and so on. A shift to sustainable food consumption patterns is seen as a necessity to achieve Sustainable Development Goals such as zero hunger, good health and wellbeing, responsible consumption and production, climate action, life below water, and life on land^14^

Despite intensive research on the performance of different dietary patterns in achieving these goals, there is a lack of research on how environmental and health outcomes of diets are jointly associated with changes in socio-economic development. Dietary transition could result in win–win solutions to health and environmental issues (for example, reducing red meat consumption lowers health risks and environmental pressures^14^) or trade-offs (for example, an increase in the consumption of dairy products can benefit human health for populations with undernutrition but adds to GHG emissions^15^). As food supply inevitably consumes non-renewable natural resources and generates pollution, knowledge is required on how efficiently these scarce environmental inputs are utilized in generating specific health benefits. Moreover, how and why such health–environment efficiency changes remains an open question. A number of studies examined environmental and nutrition/health implications of actual or hypothetical dietary patterns and discuss implications for future food policies^16–19^. Dietary patterns have been changing rapidly over time especially in recent years as low- and middle-income countries reduce undernourishment and transition to more unhealthy Western-style diets^20^, while diets in high-income countries are criticized for higher shares of red and/or processed meat, saturated fat and added sugar^21^. As a result, it is essential to examine the interconnected environmental and health outcomes of dietary changes temporally as well as how they are affected by socio-economic development.

In this Article, we explore how the environmental–health interactions of diets in all countries are associated with the socio-economic development using national-level panel data for 195 countries for the time period 1990–2011. We construct a health–environment efficiency indicator, defined as a ratio between health benefits and environmental impacts resulting from food consumption and production, to interpret how diets in various countries perform in supporting healthy lives at the cost of environmental pollution and resource consumption. Health benefits are indicated by a reduction in disability-adjusted life years (DALYs) that quantify the lost years of healthy life due to either premature mortality or disability associated with dietary intake^21,22^. Health–environment efficiency is calculated for multiple environmental pressures including GHG emissions, scarcity-weighted water withdrawal, acidifying emissions and eutrophying emissions. These four environmental pressures represent the major footprints that address different environmental issues related to diets^23^.

We investigate how this set of health–environment efficiency indicators is associated with a country’s level of development as measured by the Socio-Demographic Index (SDI). The SDI is a geometric mean of normalized per capita income, education attainment and fertility rate^24^. It is similar to the Human Development Index (HDI, calculated as the geometric mean of per capita income, education attainment and life expectancy^25^) which is frequently used in environmental footprint analysis^26,27^. However, unlike the HDI, SDI excludes direct health outcomes, thereby preventing the conflation of determinants and outcomes^28^. The SDI is commonly used for cross-country comparison of health outcomes in different socio-economic contexts^21,29^. We found that the health–environment efficiency has experienced an N-shaped change along with the increase of SDI, with the correlation between the two variables being positive (the first stage), then negative (the second stage) and finally positive again (the third stage). These results can be well explained by the multi-phase diagram of global dietary transition proposed in the literature^30^ as each of the three stages in this N-shaped curve can be mapped onto a specific phase of the global dietary transition diagram. Finally, we discuss the challenges and opportunities faced by policymaking in each phase regarding improving the health–environment efficiency. This indicator of health–environment efficiency serves as a valuable instrument for integrating health and environmental effects, and can be adopted for guiding the efforts of countries at different development levels towards dietary sustainability.

## Global dynamics of dietary environmental and health impacts

**Fig. 1:**
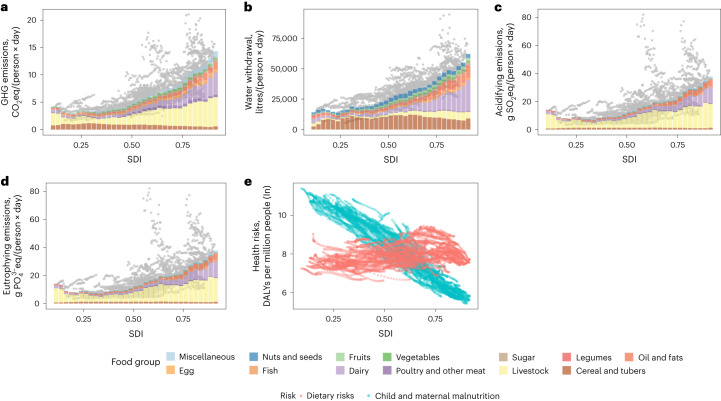
Environmental impacts and health loss of food supply in all countries at different levels of development. **a**–**e**, GHG emissions (**a**), water withdrawal (**b**), acidification (**c**), eutrophying emissions (**d**) and DALYs per million people in natural logged form (**e**). In **a**–**d**, bar charts represent average environmental impacts per country and SDI level, while the grey points represent the life-cycle environmental impact of per capita daily diets in each country. Source data

We initially examine the dynamics of environmental pressure and health impacts arising from changes in food consumption across different socio-economic development levels. Environmental impacts of food consumption have increased. As shown in Fig. 1, each environmental impact per capita correlates positively with the socio-economic development level. Environmental impacts are boosted mainly by a larger supply of animal products (particularly meat and dairy products), which are usually more resource and pollution intensive during production, while non-starchy plant-based food contributes marginally. Concurrently, there is a consistent reduction in total DALYs, primarily attributed to declining rates of child and maternal malnutrition that is linked to undernutrition and micronutrient deficiency in developing countries. Nevertheless, these benefits are offset by the quick rise of non-communicable diseases linked to suboptimal diets, characterized by over-intake of sugar, salt, red and processed meats, and other harmful dietary components but insufficient (although increasing) intake of fruits, vegetables, whole grains and other beneficial dietary components. As the development level increases, these dietary patterns increase the health risks of non-communicable diseases related to diet, including obesity, cardiovascular and cerebrovascular diseases, and diabetes^20^. This pattern shows a typical double burden of malnutrition^31^.

## Health–environment efficiency along development stages

**Fig. 2:**
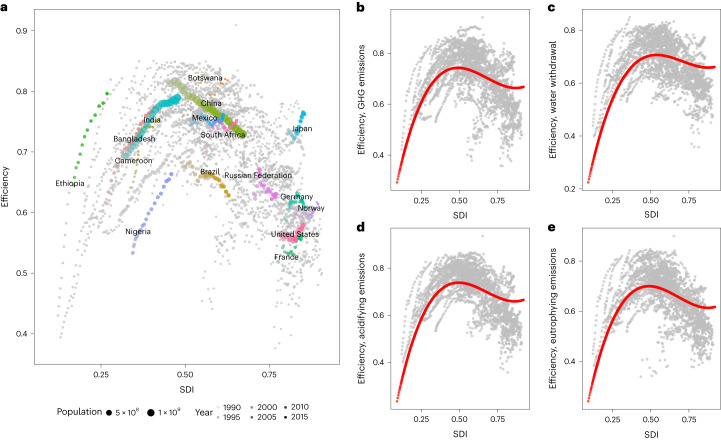
Change of dietary efficiency along socio-economic development. **a**–**e**, Total efficiency (**a**), efficiency for GHG emissions (**b**), efficiency for water withdrawal (**c**), efficiency for acidifying emissions (**d**) and efficiency for eutrophying emissions (**e**). The points show the coordination of (SDI, efficiency) for each country and year, while the red lines in **b**–**e** show the fitted cubic function based on the regression analysis. The coloured points in **a** show the trend of some countries at different stages. The detailed regression analysis for the fitted function are included in Supplementary Tables 1–4. Source data

We developed the health–environment efficiency indicator by combining environmental impacts and DALYs related to food consumption, and examined how it changes with socio-economic development across countries and over time. The health–environment efficiency indicator represents the ratio of DALYs (indicating health benefits) as numerator and a specific environmental impact in the denominator, both normalized and transformed into an exponential scale ranging between 0 and 1. In this way, lower DALY values will lead to a higher health benefit to impact the health–environment efficiency of the food system, while lower environmental impacts also result in higher efficiency indicating that natural resources are efficiently used in supporting healthy lives. This efficiency across the globe displays an N-shaped function along socio-economic development (as measured by the Socio-Demographic Index or SDI) for four environmental impacts (Fig. 2a). The health–environment efficiency rises with SDI in the least developed world in the first stage until it reaches a turning point, after which it declines in the second stage. After SDI increases to a certain level, efficiency starts to increase again in some developed countries in the third stage.

This relationship further explored using regression analysis on SDI and health–environment efficiency for each environmental impact. Multiple functional forms including the first to fourth order of SDI are examined. The regression analysis shows statistical significance of both quadratic and cubic specifications (columns 2 and 3, Supplementary Tables 1–4), while the fourth-order polynomial of SDI is not significant when introduced (column 4, Supplementary Tables 1–4). The cubic specification also yields the smallest value of Akaike information criterion (AIC) and Bayesian information criterion (BIC), along with larger adjusted within *R*^2^ (the goodness of fit excluding the fixed effects and only capturing the variation in the dependent variable within each country due to the changes in SDI). In this sense, it provides a better fit for the data compared with other functional forms (columns 1–3, Supplementary Tables 1–4).

As advanced medical care can offset some of the adverse health effect of unhealthy diets, particularly in developed countries, we introduced the Healthcare Access and Quality (HAQ) index as an additional control variable and reran the models to ensure the robustness of our findings. The significance of all terms remains after the HAQ is controlled for (columns 5–8, Supplementary Tables 1–4), indicating the robustness of the functional form of the model as well as our results. Based on the regressions, we plot the fitted value of the health–environment efficiency indicator for each environmental impact as shown in Fig. 2b–e. The turning points of the efficiency for each environmental impact are similar (SDI 0.49 and 0.87 for GHG emissions, 0.54 and 0.88 for water withdrawal, 0.50 and 0.86 for acidifying emissions, and 0.49 and 0.87 for eutrophying emissions, calculated on the basis of coefficients in column 3, Supplementary Tables 1–4). We also performed a spline regression as a non-parametric robustness check. Linear splines are adopted without penalty. The predictive accuracy of the models using a tenfold cross-validation based approach however presents a lower mean of the mean-square error for the model with two splines (Supplementary Fig. 1), indicating an inversed-U-shaped instead of an N-shaped curve as the best model choice with turning points at around SDI of 0.5 for all environmental impacts (Supplementary Table 9). In this sense, it is possible that the health–environment efficiency raises again after the decline in the second stage, but more cases are still required for this third stage to provide solid evidence of the existence of the third stage as a part of the N-shaped curve.

## Dietary transition changes health–environment efficiency

**Fig. 3:**
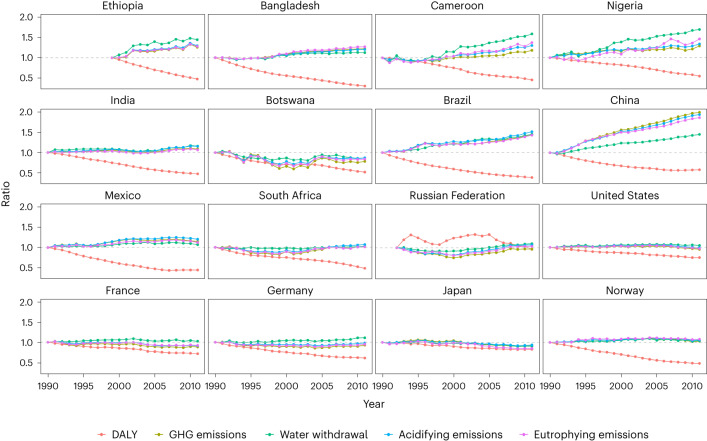
Temporal change of DALY and environmental impacts in selected countries. The value of each point connected by the lines is calculated as the ratio to the corresponding factor (DALY or environmental impacts) in the base year. Data are not available for all the countries during the time period 1990–2011 (for example, the statistics are lacking in 1990 for the Russian Federation) and thus present absence in some cases. Source data

Such multi-stage changes in efficiencies can be attributed to distinct rates of change of environmental impacts and health loss dominated by different drivers of dietary shifts, as these three stages can be mapped onto the last three phases of the dietary transition as first identified by Popkin^30^. We selected some countries with different levels of socio-demographic development and plotted the change of their DALYs and environmental impacts relative to the base year of 1990 in Fig. 3, as well as the per capita food supply by food group in Supplementary Fig. 2. In the less developed world in phase 1, famines are reduced as food security improves fuelled by increases in socio-economic development and improved agricultural productivity (for example, Ethiopia, Bangladesh, Cameroon and Nigeria). The reduced undernourishment thus boosts health benefits leading to larger resource consumption and pollution until the development level reaches the first turning point after which the efficiency declines. As socio-economic development continues, low- and middle-income countries are now experiencing a rapid dietary transition^32^, that is, towards diets containing less starchy staples and more animal products in phase 2 in Popkin’s framework of the global dietary transition accompanied by a higher intake of added sugar, high fat and high salt, as well as ultraprocessed foods (typically Brazil and China, and even continues for countries with higher SDIs such as the Russian Federation). These effects are also observed in other studies on India^33^, Brazil^34^, Egypt, Mexico and South Africa^11^. As a result, the marginal health benefits of increasing food supply become smaller as populations become more well fed while health risks for diet-related chronic diseases and environmental impacts start to increase mainly due to more consumption of animal-sourced food products. This change leads to a growing environmental impact (as the denominator) and a only marginally increasing or even shrinking health benefit (as the numerator) and leads to a decline in the health–environment efficiency until the development as measured by SDI passes the second turning point after which the health–environment efficiency increases again. Finally, for some countries such as Japan and Norway, we observed that their DALYs compared with the base year continue to decline, which might indicate that dietary patterns transit towards healthier diets in the next phase characterized with higher intake of high-quality carbohydrates and proteins, healthy fats, fruits and vegetables, and lower intake of low-quality carbohydrates due to an increased public attention to health. In this phase, red-meat consumption approaches its maximum, while the consumption of healthy foods such as legumes, fruits and vegetables further increases and thus reduce health risks, and thus the efficiencies improves as dietary health risks decline with only a slight increase in environmental impacts. However, not all developed countries have demonstrated the trend of stepping into phase 3 (for example, Germany shows a fluctuation of the health–environment efficiency in Fig. 2).

Such a transition happens alongside multiple aspects of socio-economic development, which lead to an increase in food supply and dietary diversity but also to an increase in consumption of unhealthy food^35^. Improved agricultural productivity due to technical advances increases the supply and intake of energy-intensive food such as vegetable oils. International trade openness has not only diversified the types of food but also reduced seasonal variation of food available to households^36,37^. As more women enter the labour market and traditional gender roles shift, there is a trend towards dining out rather than home cooking^38^. This shift coincides with an increase in the income of females, contributing to economic growth and advancing gender equality, which in turn impacts food affordability and allocation^39^. Easily and instantly available processed food become popular, making portability, storage and preparation easier at an affordable price^36^. However, these foods typically exhibit low nutrient density, attributed to nutrient loss during additional processing steps, along with associated environmental impacts^40,41^. By contrast, the consumption of healthy foods such as vegetable and fruits is less frequently part of daily diets as they are not only more perishable and less readily available and affordable, but are also less palatable than low-nutrient-density unhealthy foods, such as ultraprocessed foods, and thus these nutrient-dense options may not be incorporated into people’s diets in the quantities necessary for a healthy diet^42^. As countries become more developed, monetary cost still plays a key role as healthy food is usually more expensive and thus less affordable for disadvantaged groups with limited budgets^43,44^. Moreover, barriers such as high opportunity costs of preparation time play an important role in preventing individuals to pursue healthy diets^45^. The restructuring of the food environment is accelerating such change: more restaurants are readily accessible during rapid urbanization responding to the growing demand of dining out^20^. Yet food served by the food industry is usually made tasty with high fat, sodium and meat content but less vegetables to attract customers^46,47^.

## Discussions and policy implications

The proposed health–environment efficiency indicator provides a useful tool for integrating health and environmental impacts, whether they align synergistically or present contradictions. Its calculation can further be modified to tailor the focuses of management by incorporating additional, more regionally or globally crucial environmental metrics to account for a broader spectrum of ecological concerns, offering a more nuanced perspective on dietary choices. Furthermore, the health–environment efficiency indicator can be adapted to consider the temporal changes in environmental impacts driven by technological progress and other dynamic factors. This feature allows for a more comprehensive understanding of the evolving relationship between diet, health and environmental sustainability. From a policy perspective, this tool offers a critical link to existing statistical databases, making it highly practical for a range of applications. Policymakers can utilize this indicator to facilitate inter-country comparisons, helping identify best practices and areas for improvement across different nations. Moreover, it enables temporal within-country monitoring, providing a means to assess the effectiveness of policy interventions and dietary trends over time. In this way, the health–environment efficiency indicator contributes to evidence-based policymaking and the pursuit of sustainable dietary choices on both a national and global scale.

This health–environment efficiency with increasing development sheds lights on key actions for countries during different phases of Popkin’s dietary transition framework to approach the interconnected environmental and health sustainability. While prioritizing health in dietary choices is a commendable goal, it should be noted that improvements in dietary quality do not universally translate into a reduction in food-related environmental impacts, and more environmentally sustainable diets do not automatically indicate healthier options. As a result, countries should be targeting achieving healthy diets with minimized environmental impacts, which could be achieved in different ways at different stages of the N-shaped curve. For developed countries at the right end of SDI, it is possible to increase the efficiency as demonstrated by Japan and Norway, with an effective structural change of diets in both the reduction of meat consumption while increasing the intake of fruits, vegetables, whole grains and other healthy foods. The former contributes notably to lower environmental impacts given its disproportionally high emissions and resource use intensity compared with other food groups^48^. Meanwhile, increasing consumption of healthy food choices can substantially reduce DALYs in most developed countries^21^. Such health benefits come with only small additional environmental impacts that can be easily balanced out by the reduced environmental impacts from less meat consumption. In terms of policymaking, market-based approaches may play a role based on either health or environmental externalities. Empirical evidence has shown that healthy food subsidies and unhealthy food taxation can effectively change dietary behaviour^49^, while simulations also demonstrate remarkable health and environmental benefits of GHG taxes on food that can contribute to discouraging the consumption of meat^50–52^. Nudging could provide an alternative to encourage healthier, low-environmental-impact dietary patterns via carbon/nutrition labelling, changing accessibility and visibility of specific foods^53^, despite that its magnitude and long-term effects are yet to be evaluated comprehensively with more empirical studies^54^.

For low- and middle-income countries heading towards a westernization of diets and declining health–environment efficiency, guidance on dietary behaviour through education, urban planning and other measures that take long to be effective are equally if not more urgently required. Considering the inertia and path dependency of infrastructure, institutions and human behaviour unsustainable lifestyles including dietary patterns can be hard to change once cultivated within specific physical and socio-economic contexts^55^. Early stages of urbanization provide windows of opportunity for urban planning in designing food spaces with easy access to healthy foods such as vegetables and fruits, for example, community gardens, grocery stores and farmer markets accompanied with well-developed infrastructure including public and affordable transportation. On the demand side, consumer behaviour can be change resistant due to the inertia of socio-economic, cultural and physical environment contexts^56^. Behavioural habits can thus be locked in specific long-run development pathways and become difficult to change^55,56^. Immediate interventions are crucial to prevent the adoption of diets high in fat and meat content. The significance of prompt action is further underscored by the potential for increased medical costs and ecological stress caused by the large and growing population undergoing a dietary transition towards high-impact and unhealthy diets^57^. As for developing countries whose efficiency levels are still increasing largely due to the elimination of hunger, improvement of the health–environment efficiency may still predominantly come from the reduction of stunting by increasing food supply. As this requires additional inputs of environmental resources and absorptive capacity of pollution, further increase in the health–environment efficiency can come from advances in productivity. Meanwhile, precautionary guidance is required to prevent dietary mistakes from more advanced countries.

Some caveats and limitations should be noted for this study. Firstly, our regression analysis examining how development levels affect the health–environment efficiency can be affected by endogeneity issues without the inclusion of many factors, either observable or not, that affects environmental impacts and diet-related DALYs. Moreover, there can be a potential concern of reverse causality as the environmental pressure and health impacts further affect the social and economic development. This is partially alleviated via the adoption of fixed effects and robustness checks using lagged SDI as regressors (see more details in Methods), but the possibility that our estimation is confounded by missing variables remains. Specifically, the tie of this indicator to food systems is only via the environmental impacts as the denominator, whereas the health impact could be affected by factors beyond dietary change such as social inequality^58,59^ and discrimination^60^. Further studies could introduce more control variables and/or alternative model forms to improve the performance of the regression. In addition, the environmental impacts per gram of food are presented in global mean values and cover the environmental impacts during the total food supply chain from production before the food items reach the farm gate to post-farm processes such as transporting, packaging, storage and retail. A limitation of this dataset is that regional differences in terms of environmental impacts of producing the same food item is lost due to distinctions in production technologies and/or climate conditions. Moreover, it does not differentiate productivity in food production nor distance in international trade that vary by country. Nevertheless, as our focus is on changes in food consumption and their environmental and health outcomes, using global mean values helps isolate such effects from production-side differences along global supply chains. It should be noted that the dataset is based on developed countries due to better data availability, and thus may either overestimate (due to mechanized production) or underestimate (due to more energy/water efficient technologies) the environmental impacts when applied globally. Nevertheless, even though the environmental impacts of producing food varies depending on the region, the ranking of which food items have a greater impact on the environment is similar across regions, which ensures the credibility of our analysis^61^. With improved data quality and availability, these distinctions can and should be incorporated in future research. Moreover, our study focuses on the national average for food consumption and thus omits the heterogeneity within countries, which can be substantial particularly for the most populated countries and/or those with uneven development levels that warrant further exploration in future studies. Finally, as this efficiency indicator only addresses the environmental and health dimensions of sustainability as a multi-dimension concept, future studies could integrate more measurements of economic and social sustainability in this metric for more comprehensive evaluations.

## Methods

### Food supply

We retrieved per capita consumption of different food groups for each country from the Global Expanded Nutrient Supply (GENuS) database developed by Harvard University and Tufts University^62^. This dataset contains the individual daily supply of 225 food categories as well as 23 nutrients (that is, calories, fat, protein, carbohydrates, dietary fibre, vitamin C, vitamin A, folate, thiamin, riboflavin, niacin, total B6, calcium, iron, zinc, potassium, copper, magnesium, selenium, phosphorus, saturated fatty acids, monounsaturated fatty acids and polyunsaturated fatty acids) in 175 countries and regions (there are 175 countries in the introductive paper of the GENuS dataset^63^, but 15 of them are with 0 values for all the 225 food categories, and 2 are with extraordinary values that are too far away from Food and Agriculture Organization Statistics (FAOSTAT); we exclude these 17 countries from the evaluation). during the period of 1960–2011. Food supply data are constructed on the basis of the food balance sheet from FAOSTAT with a more detailed division of food categories with the non-edible portion excluded. The supply of nutrients is estimated using the food content tables from regions that each country locates in. The method of constructing the database has been validated through a comparison with the independent estimates by the United States Department of Agriculture for historical US nutrition that shows good agreement, although more validations against other countries were not possible due to limited data availability^63^. GENuS describes the food supply available for human consumption at the retail level, that is, before the food enters the household^64^, which is usually higher than the actual food intake considering the possible food waste during the household storage and food preparation. To exclude such waste, we adopt the waste ratio from the Food and Agriculture Organization^65^. These ratios provide food-group- and region-specific percentage of the food wasted in the consumption phase, each linked with the food categories in GENuS. Since the data are not available for all the country × years, the final sample contains 2,816 rather than 195 × 22 = 4,290 observations.

### Environmental impacts

We link the food supply with environmental impact factors (GHG and other impacts per gram of food) from Life Cycle Assessment (LCA) studies to quantify the life-cycle environmental impacts of dietary patterns following previous global-scale evaluations^1,48^. The LCA factors from a globally reconciled database are based on Poore and Nemecek^23^. This database collects more than 1,500 studies to summarize the GHG emissions, scarcity-weighted water withdrawal, land occupation, acidifying emissions and eutrophying emissions per gram of edible portion of 43 categories of food produced globally. These impacts represent the most critical pressure imposed by food production and consumption on the natural environment. We exclude land use from our evaluation as the impacts of land use are highly contingent upon the properties of the endogenous ecosystems that agricultural land uses replace. We then match the food categories with the items in the GENuS database to estimate the environmental impact of the food supply in each country.

### Health benefit

Health benefits are indicated by negative DALYs representing different health risks related to food consumption. The DALY quantifies the number of years lost due to ill health, disability or early death, and thus can provide a measurement of the gap between the current and ideal health status in the population of a country^66^. The data are retrieved from the Global Burden of Disease Study Database (2017). This dataset contains DALYs due to different health risks of 195 countries during the period of 1990–2017. We select DALYs related to two health risks, child and maternal malnutrition, as well as dietary risks as a measurement of health loss, which are attributable to insufficient or inadequate nutrition and dietary intakes, including both under- and over-nutrition^21,22^.

### Socio-economic development

The level of socio-economic development is indicated by the SDI developed by the Global Burden of Disease Project^24^. It is commonly used in public health studies for cross-country comparison of health outcomes^21,29^. This composite indicator averages income per person, educational attainment and total fertility rate to describe the level of development of each geography with a range from 0 to 1. Such a construction approximates other comprehensive measurements of socio-economic development level such as the HDI, which is widely applied in exploring how environmental impacts evolve with development^26,27^. Different from other indices, however, health outcomes (for example, life expectancy in HDI) are not involved in the calculation of but is regarded as affected by SDI. In this way, SDI avoids mixing up the determinant (development level) with its output (health impacts)^28^, and thus is suitable for the aim of this study.

### Evaluating the health–environment efficiency

The environmental–health association of dietary patterns is required to indicate what health benefit can be obtained with per unit of environmental input. We approximate the concept of the carbon intensity of human wellbeing^67,68^ and construct an indicator as a ratio of the two. We adopt a max–min normalization to transform the DALYs, the numerator, and the environmental impacts, the denominator, into common scales. Since DALYs indicate health loss, a transformation is required to convert it into a proxy of health benefit. As such proxy is ideally larger than 0, simply adding a negative sign to the normalized DALYs does not meet the requirement. So we calculate the exponent of both, which gives

1 $${{{\mathrm{efficiency}}}}_{{{nit}}}={e}^{-\frac{{{{\mathrm{DALY}}}}_{{{it}}}-{{{\mathrm{DALY}}}}_{\min }}{{{{\mathrm{DALY}}}}_{\max }-{{{\mathrm{DALY}}}}_{\min }}-\frac{{{{\mathrm{environment}}}}_{{{nit}}}-{{{\mathrm{environment}}}}_{{n{\mathrm{min}}}}}{{{{\mathrm{environment}}}}_{{n{\mathrm{max}}}}-{{{\mathrm{environment}}}}_{{n{\mathrm{min}}}}}}$$

in which ${{{\mathrm{environment}}}}_{{nit}}$ is the environmental impact of type $n$ (one of GHG emissions, scarcity-weighted water withdrawal, acidifying emissions and eutrophying emissions) in country *i*, year $t$. ${{{\mathrm{environment}}}}_{{n{\mathrm{min}}}}$ is the minimum value of the environmental impact among all the countries and years while ${{{\mathrm{environment}}}}_{{n{\mathrm{max}}}}$ is the maximum value. In this study we calculate the indicator for GHG emissions, scarcity-weighted water withdrawal, acidifying emissions and eutrophying emissions. Similarly, ${{{\mathrm{DALY}}}}_{{it}}$ is the DALY in country *i*, year $t$, and ${{{\mathrm{DALY}}}}_{\min }$ and ${{{\mathrm{DALY}}}}_{\max }$ are the minimum and maximum values among all the countries and years, respectively. $e$ is the universal constant. In this way, the numerator shows health benefits as a decreasing monotonic transformation of DALYs normalized by its range of the whole sample, while the denominator is a positive monotonic transformation of the averaged normalized environmental impacts. A larger value indicating a better performance of diets in environmental and health sustainability (that is, larger health benefits but smaller environmental impacts). This functional form is characterized by the following useful properties: First, the health–environment efficiency is a monotonic decline of both DALYs and each type of environmental impact that fits in the concept that we are targeting; second, all the impacts are normalized to be comparable; third, it provides a positive number ranging from *e*^−^^2^ to 1.

### Statistical analysis

We conduct a regression analysis to test the correlation between SDI and the efficiencies based on each environmental impact. We test different functional forms including the linear, quadratic and cubic settings, and select the model of best fit based on the value of both AIC and BIC. We adopt the generalized least square strategy using a fixed-effect model as follow (taking the cubic function as an example):

2 $${{{\mathrm{efficiency}}}}_{{nit}}={\beta }_{1}{{{\mathrm{SDI}}}}_{{it}}+{\beta }_{2}{{{{\mathrm{SDI}}}}_{{it}}}^{2}+{\beta }_{3}{{{{\mathrm{SDI}}}}_{{it}}}^{3}+{\alpha }_{i}+{\lambda }_{t}+{\varepsilon }_{{it}}$$

where ${{{\mathrm{SDI}}}}_{{it}}$ denotes the SDI of country *i* in year $t$. ${\alpha }_{i}$ is the country fixed effect and ${\lambda }_{t}$ is the year fixed effect. ${\varepsilon }_{{it}}$ is the error term.

There might be endogeneity issues due to missing variables since SDI and the health–environment efficiency indicator can be simultaneously affected by unobservable variables that are not controlled for. We thus introduce country fixed effect and year fixed effect to control for time-invariant attributes of a countries and a common time trend, respectively, that could be correlated with both SDI and the health–environment efficiency indicator. In this way, the missing variable issue can be at least partly alleviated. To further confirm the temporal precedence of SDI in advance of the health–environment efficiency for potential causality against endogeneity because of reverse causality, we also rerun the regressions using SDI in the past year as a robustness check. Moreover, SDI may not be a perfect indicator for socio-economic development and thus involves an attenuation bias due to measurement error (that is, coefficients become biased towards 0). However, we are still able to obtain conservative estimates on the relationship between SDI and the health–environment efficiency.

The main results are presented in columns 1–3, Supplementary Tables 1–4. As the quality of health care also affects DALYs and thus the health–environment efficiency, we control for the HAQ index and rerun all the models as a robustness check, with the results presented in columns 4–6, Supplementary Tables 1–4. Developed by the Global Burden of Disease Studies, the HAQ measures the accessibility to quality health care^69^, which is used as an important health-related control variable. This indicator is available every 5 years (that is, available in 1990, 1995, 2000, 2005 and 2010) so that the sample size is smaller than the full sample. The estimates, despite being conservative due to potential measurement error, still present statistical significance with decent magnitude, indicating the correlation between SDI and the efficiency indicator. The results of the robustness check on the lagged SDI are presented in Supplementary Tables 5–8 in which the significance and magnitude of the coefficients are similar to the results in Supplementary Tables 1–4.

To testify whether the quadratic or the cubic function is the best functional form of the regression, we also conducted a spline regression as follows:

3 $${{{\mathrm{efficiency}}}}_{{nit}}=\mathop{\sum}\nolimits_{j}{\beta }_{j}\;{f}_{j}({{{\mathrm{SDI}}}}_{{it}})+{\alpha }_{i}+{\lambda }_{t}+{\varepsilon }_{{it}}$$

where the functions ${f}_{j}()$ define the splines with $j$ splines. We use equal intervals to separate linear splines (for example, there are two splines with SDI <0.5 and SDI ≥0.5 when *j* = 2, three splines with SDI <0.33, 0.33 ≤ SDI ≤ 0.67, and SDI ≥0.67 when *j* = 3, and so on) with no penalties implemented. A tenfold Cross-Validation (CV) is adopted to determine $j$. Equation (3) is trained using nine out of ten randomly split folds of the sample for different values of $j$ and predicted using the remaining one fold. The mean of the mean-square error for each prediction is calculated as CV statistics, the lowest value of which identifies the optimal *j* (ref. ^70^). We randomize the split of sample with 1,000 trials for each value of $j$ ranging from 2 to 14 in case there are more splines, and summarize the CV statistics in Supplementary Fig. 1. The results show that the model with two splines perform best in lowering the cross-validation error, indicating an inverse-U-shaped curve of the efficiency–SDI correlations. We provide the regression results in Supplementary Table 9.

### Reporting summary

Further information on research design is available in the Nature Portfolio Reporting Summary linked to this article.

### Supplementary information

Supplementary Information Supplementary Figs. 1 and 2 and Tables 1–9.

Reporting Summary

### Source data

Source Data Fig. 1 Statistical source data.

Source Data Fig. 2 Statistical source data.

Source Data Fig. 3 Statistical source data.

## Data Availability

All the data used in this study are publicly available. The GENuS database is publicly available at https://dataverse.harvard.edu/dataverse/GENuS. The FAOSTAT food balance sheet comes from http://www.fao.org/faostat/en/#data. The DALY data can be retrieved from the website of Global Burden of Disease Study Database at http://ghdx.healthdata.org/gbd-results-tool. The SDI comes from http://ghdx.healthdata.org/record/ihme-data/gbd-2015-socio-demographic-index-sdi-1980%E2%80%932015. HAQ index comes from http://ghdx.healthdata.org/record/ihme-data/gbd-2016-healthcare-access-and-quality-index-1990-2016. The LCA database is provided by Poore and Nemecek in their paper entitled ‘Reducing food’s environmental impacts through producers and consumers’ (10.1126/science.aaq0216). The data from these sources processed for this paper that can be directly applied to the coding script are also available from https://github.com/hepannju/Health-environment-efficiency-of-diets-shows-non-linear-trends-over-1990-2011.git. Source data are provided with this paper.

## Code availability

The data used in this study were processed using RStudio (based on R 3.6.1). The regression analysis are conducted in Stata/SE 17. Coding scripts are available from https://github.com/hepannju/Health-environment-efficiency-of-diets-shows-non-linear-trends-over-1990-2011.git.
