# Supplementary material for: Health–environment efficiency of diets shows nonlinear trends over 1990–2011
Source: Nat Food. 2024 Feb 8;5(2):116–24. doi: 10.1038/s43016-024-00924-z (PMC10896724; doi:10.1038/s43016-024-00924-z)
Supplement: Supplementary file 1 — Supplementary Figs. 1 and 2 and Tables 1–9. [file 43016_2024_924_MOESM1_ESM.pdf]

nature food

Article

https://doi.org/10.1038/s43016-024-00924-z

Health–environment efficiency of diets shows nonlinear trends over 1990–2011

In the format provided by the authors and unedited

Table of Supplementary Figures

Figure S1 CV statistics of the ten-fold cross validation for the spline regression. 2
Figure S2 The temporal change of per capita food supply in selected countries 2

Table of Supplementary Tables

Table S1 Regression results of the health-environment efficiency on SDI, GHG emissions 3
Table S2 Regression results of the health-environment efficiency on SDI, water withdrawal 3
Table S3 Regression results of the health-environment efficiency on SDI, acidifying emissions 4
Table S4 Regression results of the health-environment efficiency on SDI, eutrophying emissions 4
Table S5 Regression results of the health-environment efficiency on lagged SDI, GHG emissions 5
Table S6 Regression results of the health-environment efficiency on lagged SDI, water withdrawal 6
Table S7 Regression results of the health-environment efficiency on lagged SDI, acidifying emissions 6
Table S8 Regression results of the health-environment efficiency on lagged SDI, eutrophying emissions 7
Table S9 Results of spline regression with 2 splines 7

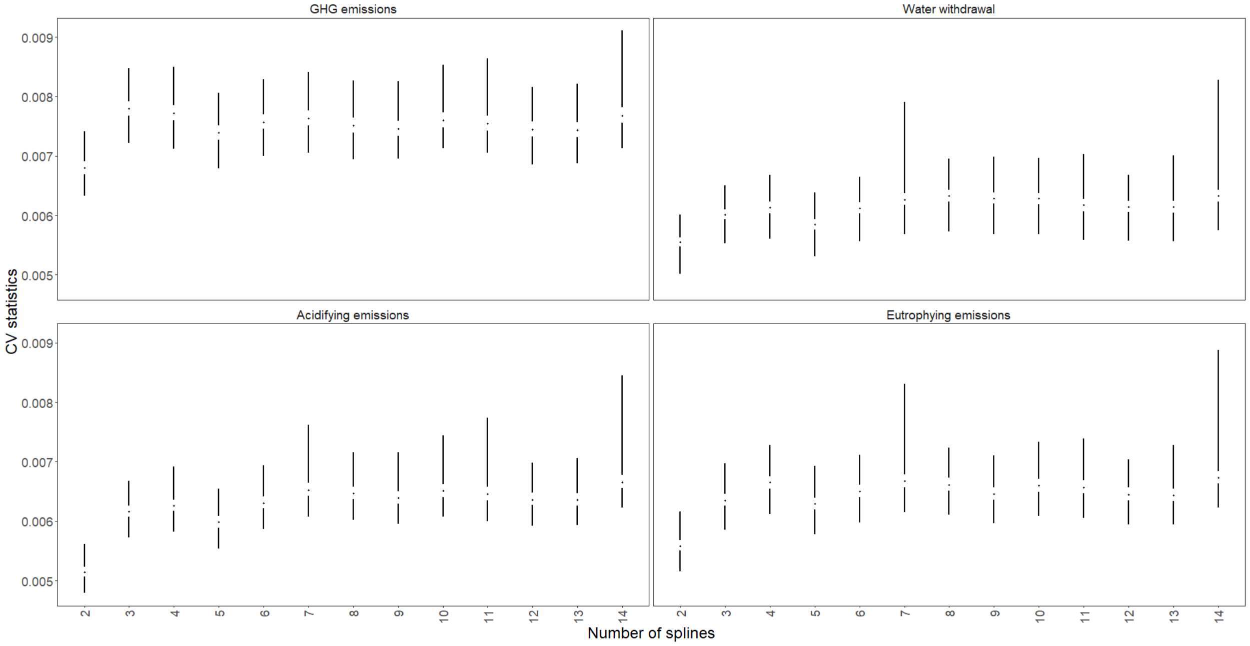

Figure S1 Tufte's Box Plot for CV statistics of the ten-fold cross validation for the spline regression. *a) GHG emissions, b) Water withdrawal, c) Acidifying emissions, d) Eutropying emissions. Linear splines are adopted without penalty. For each number of splines, 1000 trials are run for splitting the sample into ten folds. The central box denotes the interquartile range (IQR), the lower and upper edges representing the first (Q1) and third (Q3) quartiles, and the understated whiskers extend to the 10th and 90th percentiles, respectively.*

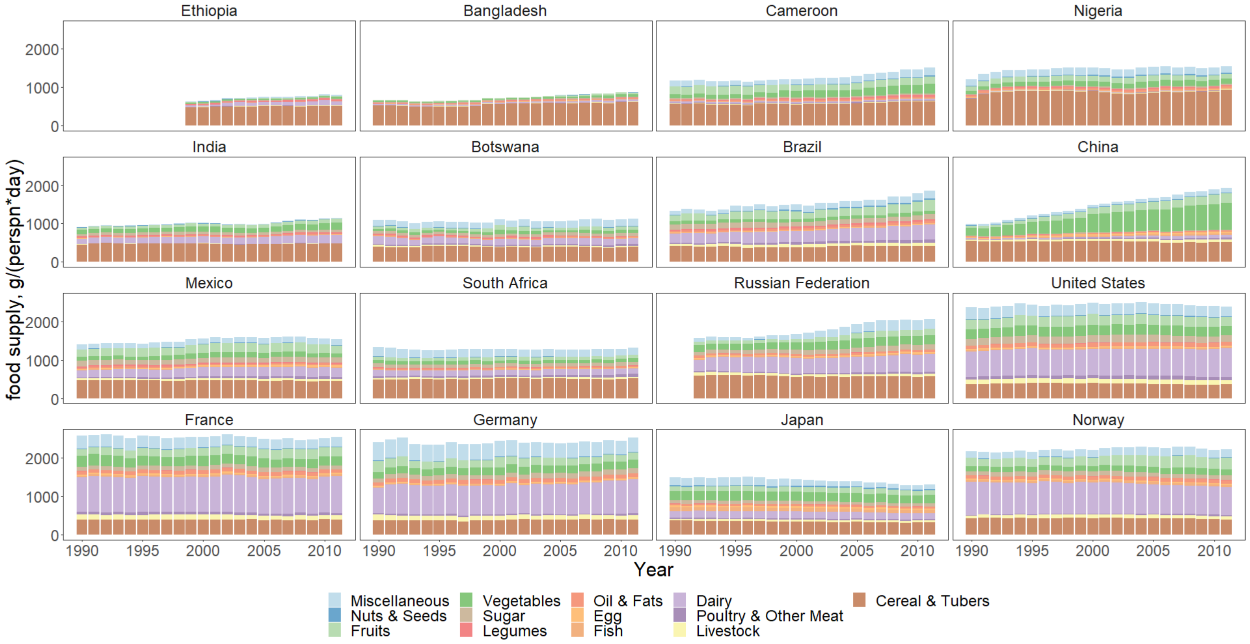

Figure S2 The temporal change of per capita food supply in selected countries. *Data are not available for all the countries during 1990-2011 (e.g. the statistics are lacking in 1990 for Russian Federation) and thus present absence in some cases.*

Table S1 Regression results of the health-environment efficiency on SDI, GHG emissions

	(1) Efficiency	(2) Efficiency	(3) Efficiency	(4) Efficiency	(5) Efficiency	(6) Efficiency	(7) Efficiency	(8) Efficiency
SDI	0.023	1.153***	3.759***	5.046***	0.167	1.122***	3.856***	4.907***
	(0.128)	(0.194)	(0.453)	(0.935)	(0.140)	(0.192)	(0.445)	(1.040)
SDI^2		-1.222***	-6.005***	-10.297***		-1.211***	-6.184***	-9.705***
		(0.196)	(0.792)	(2.957)		(0.208)	(0.767)	(3.231)
SDI^3			2.950***	8.690**			3.090***	7.830*
			(0.486)	(3.879)			(0.480)	(4.223)
SDI^4				-2.677				-2.219
				(1.802)				(1.947)
HAQ					-0.002	0.000	0.000	0.000
					(0.001)	(0.001)	(0.001)	(0.001)
Fixed effect								
Country	Y	Y	Y	Y	Y	Y	Y	Y
Year	Y	Y	Y	Y	Y	Y	Y	Y
N	2816	2816	2816	2816	624	624	624	624
Within R^2	0.000	0.189	0.274	0.279	0.011	0.204	0.311	0.314
Adjusted within R^2	-0.000	0.188	0.274	0.277	0.006	0.199	0.305	0.307
AIC	-12040.573	-12627.072	-12939.633	-12953.512	-2630.058	-2764.186	-2851.882	-2852.755
BIC	-12034.630	-12615.186	-12921.804	-12929.740	-2621.185	-2750.878	-2834.137	-2830.574

Notes: Standard errors in parentheses are clustered to the country level. Two-sided t-tests are performed for hypothesis tests. * p<0.1, ** p<0.05, *** p<0.01. Since HAQ is only available for limited years, the sample size is smaller for column 5-8. The within R^2 and adjusted within R^2 are reported which captures the goodness of fit of the regressors excluding the country and year fixed effects.

Table S2 Regression results of the health-environment efficiency on SDI, water withdrawal

	(1) Efficiency	(2) Efficiency	(3) Efficiency	(4) Efficiency	(5) Efficiency	(6) Efficiency	(7) Efficiency	(8) Efficiency
SDI	0.178	1.350***	3.581***	4.617***	0.262**	1.263***	3.559***	4.299***
	(0.112)	(0.170)	(0.379)	(0.803)	(0.114)	(0.157)	(0.377)	(0.861)
SDI^2		-1.268***	-5.362***	-8.817***		-1.270***	-5.448***	-7.928***
		(0.169)	(0.646)	(2.497)		(0.169)	(0.646)	(2.672)
SDI^3			2.526***	7.146**			2.596***	5.934*
			(0.379)	(3.231)			(0.388)	(3.488)
SDI^4				-2.155				-1.563
				(1.479)				(1.614)
HAQ					-0.001	0.001	0.001	0.001
					(0.001)	(0.001)	(0.001)	(0.001)
Fixed effect								
Country	Y	Y	Y	Y	Y	Y	Y	Y
Year	Y	Y	Y	Y	Y	Y	Y	Y
N	2816	2816	2816	2816	624	624	624	624
Within R^2	0.010	0.292	0.379	0.382	0.018	0.309	0.411	0.413

Adjusted within R^2	0.010	0.291	0.378	0.382	0.014	0.305	0.406	0.407
AIC	-12991.229	-13930.946	-14298.892	-14313.564	-2827.846	-3044.836	-3142.880	-3143.151
BIC	-12985.286	-13919.060	-14281.062	-14289.791	-2818.974	-3031.528	-3125.135	-3120.971

Notes: Standard errors in parentheses are clustered to the country level. Two-sided t-tests are performed for hypothesis tests. * p<0.1, ** p<0.05, *** p<0.01. Since HAQ is only available for limited years, the sample size is smaller for column 5-8. The within R^2 and adjusted within R^2 are reported which captures the goodness of fit of the regressors excluding the country and year fixed effects.

Table S3 Regression results of the health-environment efficiency on SDI, acidifying emissions

	(1) Efficiency	(2) Efficiency	(3) Efficiency	(4) Efficiency	(5) Efficiency	(6) Efficiency	(7) Efficiency	(8) Efficiency
SDI	0.043	1.283***	4.164***	5.023***	0.166	1.218***	4.214***	4.900***
	(0.125)	(0.198)	(0.425)	(0.906)	(0.134)	(0.191)	(0.430)	(0.991)
SDI^2		-1.341***	-6.626***	-9.494***		-1.335***	-6.785***	-9.084***
		(0.198)	(0.732)	(2.866)		(0.208)	(0.728)	(3.077)
SDI^3			3.260***	7.095*			3.386***	6.482
			(0.441)	(3.745)			(0.447)	(4.005)
SDI^4				-1.788				-1.450
				(1.726)				(1.838)
HAQ					-0.001	0.001	0.001	0.001
					(0.001)	(0.001)	(0.001)	(0.001)
Fixed effect								
Country	Y	Y	Y	Y	Y	Y	Y	Y
Year	Y	Y	Y	Y	Y	Y	Y	Y
N	2816	2816	2816	2816	624	624	624	624
Within R^2	0.000	0.254	0.371	0.373	0.009	0.262	0.399	0.401
Adjusted within R^2	0.000	0.253	0.370	0.372	0.005	0.258	0.394	0.395
AIC	-12350.800	-13171.186	-13648.917	-13656.023	-2673.597	-2855.565	-2982.067	-2981.576
BIC	-12344.857	-13159.300	-13631.088	-13632.250	-2664.725	-2842.257	-2964.323	-2959.395

Notes: Standard errors in parentheses are clustered to the country level. Two-sided t-tests are performed for hypothesis tests. * p<0.1, ** p<0.05, *** p<0.01. Since HAQ is only available for limited years, the sample size is smaller for column 5-8. The within R^2 and adjusted within R^2 are reported which captures the goodness of fit of the regressors excluding the country and year fixed effects.

Table S4 Regression results of the health-environment efficiency on SDI, eutrophying emissions

	(1) Efficiency	(2) Efficiency	(3) Efficiency	(4) Efficiency	(5) Efficiency	(6) Efficiency	(7) Efficiency	(8) Efficiency
SDI	0.017	1.185***	3.841***	4.589***	0.136	1.138***	3.830***	4.592***
	(0.131)	(0.198)	(0.441)	(0.914)	(0.140)	(0.190)	(0.433)	(0.993)
SDI^2		-1.264***	-6.136***	-8.631***		-1.271***	-6.169***	-8.722***
		(0.196)	(0.773)	(2.871)		(0.203)	(0.749)	(3.083)
SDI^3			3.006***	6.342*			3.043***	6.481
			(0.471)	(3.730)			(0.463)	(4.012)
SDI^4				-1.556				-1.610
				(1.716)				(1.842)

HAQ					-0.002	0.001	0.000	0.000
					(0.001)	(0.001)	(0.001)	(0.001)
Fixed effect								
Country	Y	Y	Y	Y	Y	Y	Y	Y
Year	Y	Y	Y	Y	Y	Y	Y	Y
N	2816	2816	2816	2816	624	624	624	624
Within R^2	0.000	0.204	0.294	0.295	0.009	0.227	0.332	0.333
Adjusted within R^2	-0.000	0.203	0.293	0.294	0.005	0.222	0.326	0.326
AIC	-12070.502	-12709.721	-13045.422	-13048.981	-2640.030	-2792.656	-2881.790	-2881.374
BIC	-12064.559	-12697.835	-13027.593	-13025.209	-2631.157	-2779.348	-2864.045	-2859.193

Notes: Standard errors in parentheses are clustered to the country level. Two-sided t-tests are performed for hypothesis tests. * p<0.1, ** p<0.05, *** p<0.01. Since HAQ is only available for limited years, the sample size is smaller for column 5-8. The within R^2 and adjusted within R^2 are reported which captures the goodness of fit of the regressors excluding the country and year fixed effects.

Table S5 Regression results of the health-environment efficiency on lagged SDI, GHG emissions

	(1) Efficiency	(2) Efficiency	(3) Efficiency	(4) Efficiency	(5) Efficiency	(6) Efficiency	(7) Efficiency	(8) Efficiency
SDI	0.028	1.120***	3.860***	5.404***	0.018	1.038***	4.117***	4.852***
	(0.125)	(0.197)	(0.466)	(0.948)	(0.161)	(0.206)	(0.486)	(1.238)
SDI^2		-1.157***	-6.209***	-11.367***		-1.202***	-6.843***	-9.290**
		(0.196)	(0.814)	(2.991)		(0.215)	(0.879)	(3.806)
SDI^3			3.113***	10.034**			3.496***	6.771
			(0.501)	(3.928)			(0.549)	(4.917)
SDI^4				-3.236*				-1.526
				(1.827)				(2.260)
HAQ					0.000	0.002	0.001	0.001
					(0.001)	(0.001)	(0.001)	(0.001)
Fixed effect								
Country	Y	Y	Y	Y	Y	Y	Y	Y
Year	Y	Y	Y	Y	Y	Y	Y	Y
N	2718	2718	2718	2718	526	526	526	526
Within R^2	0.000	0.168	0.262	0.268	0.000	0.181	0.308	0.309
Adjusted within R^2	-0.000	0.167	0.261	0.267	-0.005	0.175	0.300	0.300
AIC	-11761.399	-12259.041	-12582.774	-12602.259	-2364.155	-2467.306	-2553.467	-2552.532
BIC	-11755.492	-12247.226	-12565.051	-12578.628	-2355.624	-2454.511	-2536.406	-2531.206

Notes: Standard errors in parentheses are clustered to the country level. Two-sided t-tests are performed for hypothesis tests. * p<0.1, ** p<0.05, *** p<0.01. Since HAQ is only available for limited years, the sample size is smaller for column 5-8. The within R^2 and adjusted within R^2 are reported which captures the goodness of fit of the regressors excluding the country and year fixed effects.

Table S6 Regression results of the health-environment efficiency on lagged SDI, water withdrawal

	(1)<br>Efficiency	(2)<br>Efficiency	(3)<br>Efficiency	(4)<br>Efficiency	(5)<br>Efficiency	(6)<br>Efficiency	(7)<br>Efficiency	(8)<br>Efficiency
SDI	0.170	1.326***	3.700***	4.989***	0.074	1.150***	3.898***	4.642***
	(0.110)	(0.175)	(0.386)	(0.800)	(0.135)	(0.190)	(0.417)	(0.961)
SDI^2		-1.224***	-5.601***	-9.909***		-1.268***	-6.303***	-8.778***
		(0.172)	(0.655)	(2.495)		(0.195)	(0.778)	(3.034)
SDI^3			2.698***	8.476***			3.120***	6.433
			(0.385)	(3.242)			(0.492)	(4.031)
SDI^4				-2.702*				-1.544
				(1.490)				(1.911)
HAQ					0.001	0.002***	0.002**	0.002**
					(0.001)	(0.001)	(0.001)	(0.001)
Fixed effect								
Country	Y	Y	Y	Y	Y	Y	Y	Y
Year	Y	Y	Y	Y	Y	Y	Y	Y
N	2718	2718	2718	2718	526	526	526	526
Within R^2	0.010	0.270	0.367	0.373	0.008	0.277	0.411	0.413
Adjusted within R^2	0.009	0.269	0.366	0.372	0.003	0.271	0.405	0.405
AIC	-12668.118	-13493.650	-13881.208	-13903.371	-2519.538	-2683.733	-2789.660	-2789.369
BIC	-12662.210	-13481.835	-13863.485	-13879.741	-2511.008	-2670.937	-2772.599	-2768.042

Notes: Standard errors in parentheses are clustered to the country level. Two-sided t-tests are performed for hypothesis tests. * $p<0.1$, ** $p<0.05$, *** $p<0.01$. Since HAQ is only available for limited years, the sample size is smaller for column 5-8. The within R^2 and adjusted within R^2 are reported which captures the goodness of fit of the regressors excluding the country and year fixed effects.

Table S7 Regression results of the health-environment efficiency on lagged SDI, acidifying emissions

	(1)<br>Efficiency	(2)<br>Efficiency	(3)<br>Efficiency	(4)<br>Efficiency	(5)<br>Efficiency	(6)<br>Efficiency	(7)<br>Efficiency	(8)<br>Efficiency
SDI	0.046	1.250***	4.291***	5.383***	-0.006	1.119***	4.516***	4.910***
	(0.121)	(0.202)	(0.435)	(0.908)	(0.155)	(0.210)	(0.467)	(1.169)
SDI^2		-1.275***	-6.881***	-10.530***		-1.325***	-7.551***	-8.861**
		(0.200)	(0.747)	(2.875)		(0.220)	(0.839)	(3.610)
SDI^3			3.455***	8.350**			3.858***	5.611
			(0.451)	(3.764)			(0.526)	(4.670)
SDI^4				-2.289				-0.817
				(1.739)				(2.149)
HAQ					0.001	0.002**	0.002*	0.002*
					(0.001)	(0.001)	(0.001)	(0.001)
Fixed effect								
Country	Y	Y	Y	Y	Y	Y	Y	Y
Year	Y	Y	Y	Y	Y	Y	Y	Y
N	2718	2718	2718	2718	526	526	526	526
Within R^2	0.001	0.229	0.359	0.362	0.001	0.240	0.407	0.407

Adjusted within R^2	0.000	0.229	0.358	0.361	-0.004	0.234	0.401	0.400
AIC	-12071.823	-12775.838	-13274.394	-13286.239	-2407.407	-2549.090	-2677.511	-2675.897
BIC	-12065.915	-12764.022	-13256.671	-13262.609	-2398.877	-2536.294	-2660.449	-2654.570

Notes: Standard errors in parentheses are clustered to the country level. Two-sided t-tests are performed for hypothesis tests. * p<0.1, ** p<0.05, *** p<0.01. Since HAQ is only available for limited years, the sample size is smaller for column 5-8. The within R^2 and adjusted within R^2 are reported which captures the goodness of fit of the regressors excluding the country and year fixed effects.

Table S8 Regression results of the health-environment efficiency on lagged SDI, eutrophying emissions

	(1) Efficiency	(2) Efficiency	(3) Efficiency	(4) Efficiency	(5) Efficiency	(6) Efficiency	(7) Efficiency	(8) Efficiency
SDI	0.028	1.150***	3.970***	4.957***	-0.007	1.035***	4.132***	4.792***
	(0.127)	(0.203)	(0.455)	(0.925)	(0.165)	(0.212)	(0.474)	(1.164)
SDI^2		-1.188***	-6.388***	-9.684***		-1.227***	-6.903***	-9.099**
		(0.199)	(0.797)	(2.902)		(0.221)	(0.859)	(3.584)
SDI^3			3.205***	7.627**			3.517***	6.456
			(0.487)	(3.772)			(0.534)	(4.616)
SDI^4				-2.068				-1.369
				(1.738)				(2.112)
HAQ					0.000	0.002	0.001	0.001
					(0.001)	(0.001)	(0.001)	(0.001)
Fixed effect								
Country	Y	Y	Y	Y	Y	Y	Y	Y
Year	Y	Y	Y	Y	Y	Y	Y	Y
N	2718	2718	2718	2718	526	526	526	526
Within R^2	0.000	0.179	0.280	0.282	0.000	0.194	0.324	0.326
Adjusted within R^2	-0.000	0.179	0.279	0.281	-0.005	0.187	0.317	0.317
AIC	-11792.761	-12327.368	-12681.174	-12688.247	-2375.986	-2486.963	-2578.021	-2576.919
BIC	-11786.854	-12315.553	-12663.451	-12664.617	-2367.455	-2474.167	-2560.960	-2555.593

Notes: Standard errors in parentheses are clustered to the country level. Two-sided t-tests are performed for hypothesis tests. * p<0.1, ** p<0.05, *** p<0.01. Since HAQ is only available for limited years, the sample size is smaller for column 5-8. The within R^2 and adjusted within R^2 are reported which captures the goodness of fit of the regressors excluding the country and year fixed effects.

Table S9 Results of spline regression with 2 splines

	(1) Efficiency, GHG emissions	(2) Efficiency, water withdrawal	(3) Efficiency, acidifying emissions	(4) Efficiency, eutrophying emissions
sdi_index_value_1	0.442***	0.586***	0.496***	0.451***
	(0.136)	(0.121)	(0.140)	(0.136)
sdi_index_value_2	-0.812***	-0.792***	-0.878***	-0.842***
	(0.126)	(0.117)	(0.133)	(0.126)
Fixed effect				

Country	Y	Y	Y	Y
Year	Y	Y	Y	Y
N	2816	2816	2816	2816
Within R^2	0.182	0.250	0.238	0.198
Adjusted within R^2	0.181	0.250	0.237	0.197
AIC	-12603.851	-13772.007	-13111.952	-12688.238
BIC	-12591.965	-13760.120	-13100.066	-12676.352

Notes: Linear splines are adopted without penalty. Standard errors in parentheses are clustered to the country level. Two-sided t-tests are performed for hypothesis tests. * $p<0.1$, ** $p<0.05$, *** $p<0.01$. The within R^2 and adjusted within R^2 are reported which captures the goodness of fit of the regressors excluding the country and year fixed effects.
