# Supplementary material for: Health–environment efficiency of diets shows nonlinear trends over 1990–2011
Source: Nat Food. 2024 Feb 8;5(2):116–24. doi: 10.1038/s43016-024-00924-z (PMC10896724; doi:10.1038/s43016-024-00924-z)
Supplement: Supplementary file 2 — Reporting Summary [file 43016_2024_924_MOESM2_ESM.pdf]

nature portfolio

Corresponding author(s): Pan He, Zhu Liu

Last updated by author(s): Jan 9, 2024

Reporting Summary

Nature Portfolio wishes to improve the reproducibility of the work that we publish. This form provides structure for consistency and transparency in reporting. For further information on Nature Portfolio policies, see our Editorial Policies and the Editorial Policy Checklist.

Statistics

For all statistical analyses, confirm that the following items are present in the figure legend, table legend, main text, or Methods section.

n/a	Confirmed	
☐	☒	The exact sample size (*n*) for each experimental group/condition, given as a discrete number and unit of measurement
☒	☐	A statement on whether measurements were taken from distinct samples or whether the same sample was measured repeatedly
☐	☒	The statistical test(s) used AND whether they are one- or two-sided<br>*Only common tests should be described solely by name; describe more complex techniques in the Methods section.*
☐	☒	A description of all covariates tested
☐	☒	A description of any assumptions or corrections, such as tests of normality and adjustment for multiple comparisons
☐	☒	A full description of the statistical parameters including central tendency (e.g. means) or other basic estimates (e.g. regression coefficient) AND variation (e.g. standard deviation) or associated estimates of uncertainty (e.g. confidence intervals)
☐	☒	For null hypothesis testing, the test statistic (e.g. *F*, *t*, *r*) with confidence intervals, effect sizes, degrees of freedom and *P* value noted<br>*Give P values as exact values whenever suitable.*
☒	☐	For Bayesian analysis, information on the choice of priors and Markov chain Monte Carlo settings
☒	☐	For hierarchical and complex designs, identification of the appropriate level for tests and full reporting of outcomes
☒	☐	Estimates of effect sizes (e.g. Cohen's *d*, Pearson's *r*), indicating how they were calculated

Our web collection on statistics for biologists contains articles on many of the points above.

Software and code

Policy information about availability of computer code

Data collection	n/a - the data is prepared and does not require additional collection. No software is used for data collection.
Data analysis	The data used in this study were processed using R studio (based on R 3.6.1). The regression analysis are conducted in Stata/SE 17. Coding scripts are available from https://github.com/hepannju/Health-environment-efficiency-of-diets-shows-non-linear-trends-over-1990-2011.git.

For manuscripts utilizing custom algorithms or software that are central to the research but not yet described in published literature, software must be made available to editors and reviewers. We strongly encourage code deposition in a community repository (e.g. GitHub). See the Nature Portfolio guidelines for submitting code & software for further information.

Data

Policy information about availability of data

All manuscripts must include a data availability statement. This statement should provide the following information, where applicable:

- Accession codes, unique identifiers, or web links for publicly available datasets
- A description of any restrictions on data availability
- For clinical datasets or third party data, please ensure that the statement adheres to our policy

All the data used in this study are publicly available. The GENuS database is publicly available at https://dataverse.harvard.edu/dataverse/GENuS. The FAOSTAT food balance sheet comes from http://www.fao.org/faostat/en/#data. The DALY data can be retrieved from the website of Global Burden of Disease Study Database at http://ghdx.healthdata.org/gbd-results-tool. The Socio-demographic Index (SDI) comes from http://ghdx.healthdata.org/record/ihme-data/gbd-2015-socio-

demographic-index-sdi-1980%E2%80%932015. Healthcare Access and Quality (HAQ) Index comes from http://ghdx.healthdata.org/record/ihme-data/gbd-2016-healthcare-access-and-quality-index-1990-2016. The LCA database is provided by Poore and Nemecek in their paper entitled “Reducing food’s environmental impacts through producers and consumers” (doi: 10.1126/science.aaq0216).

Human research participants

Policy information about studies involving human research participants and Sex and Gender in Research.

Reporting on sex and gender	n/a - The data adopted for analysis are at national-level and have covered all the age and sex groups.
Population characteristics	n/a - See above.
Recruitment	n/a - the data is prepared and does not require additional collection.
Ethics oversight	n/a - the data is prepared and does not require additional collection.

Note that full information on the approval of the study protocol must also be provided in the manuscript.

Field-specific reporting

Please select the one below that is the best fit for your research. If you are not sure, read the appropriate sections before making your selection.

☐ Life sciences ☒ Behavioural & social sciences ☐ Ecological, evolutionary & environmental sciences

For a reference copy of the document with all sections, see nature.com/documents/nr-reporting-summary-flat.pdf

Behavioural & social sciences study design

All studies must disclose on these points even when the disclosure is negative.

Study description	This study examines how the environmental and health impacts of food consumption have been changing in global countries during 1990-2011. We quantify the greenhouse gas emissions, water consumption, land occupation, acidifying emissions, and eutrophying emissions related to food supply in each country, and link these data with the Disability Adjusted Life Years (DALY) related to dietary risks and malnutrition. Based on the match, we construct an indicator to evaluate how efficient each country is in providing diets of high health benefit but low environmental impacts. Finally, we analyze how this efficiency change with the socio-economic development level.
Research sample	As we focus on analysis of each country, the data come from several national-level databases instead of being collected using a sampling strategy. We combine several datasets and the final sample for analysis covers 195 global countries. The data source for each is listed below: The GENuS database is publicly available at https://dataverse.harvard.edu/dataverse/GENuS. The FAOSTAT food balance sheet comes from http://www.fao.org/faostat/en/#data. The DALY data can be retrieved from the website of Global Burden of Disease Study Database at http://ghdx.healthdata.org/gbd-results-tool. The Socio-demographic Index (SDI) comes from http://ghdx.healthdata.org/record/ihme-data/gbd-2015-socio-demographic-index-sdi-1980%E2%80%932015. Healthcare Access and Quality (HAQ) Index comes from http://ghdx.healthdata.org/record/ihme-data/gbd-2016-healthcare-access-and-quality-index-1990-2016. The LCA database is provided by Poore and Nemecek in their paper entitled “Reducing food’s environmental impacts through producers and consumers” (doi: 10.1126/science.aaq0216). To our knowledge, this database present the best sample that we can obtain for global-level representativeness that has been justified via peer review and adopted in many studies concerning the quantification of environmental impacts of food products.
Sampling strategy	We are not using a sampling strategy but conduct analysis based on existing statistics and databases. The FAOSTAT food balance sheet is construct based on reported statistics from each country using the method specified in a handbook provided by FAO (http://www.fao.org/3/x9892e/X9892E00.htm). the other data are reproduced based on the existing statistics or studies. The methods are specified in different papers listed as below. The construction of GENuS is introduced in a paper entitled "Global Expanded Nutrient Supply (GENuS) Model: A New Method for Estimating the Global Dietary Supply of Nutrients". The calculation of SDI is introduced in a paper entitled "Measuring the health-related Sustainable Development Goals in 188 countries: a baseline analysis from the Global Burden of Disease Study 2015". The construction of HAQ is introduced in a paper entitled "Healthcare Access and Quality Index based on mortality from causes amenable to personal health care in 195 countries and territories, 1990–2015: a novel analysis from the Global Burden of Disease Study 2015". Based on these databases, a sample that covers as many countries globally as possible is adopted for the study to reflect the dietary transition of the majority of global population.
Data collection	The data are retrieved from publicly available datasets with the data collection process stated in details in the handbook and the papers listed above (see "sampling strategy"). The data are stored in excel files and analyzed in R Studio and Stata. This study presents a exploratory work of the data so that the researchers do not propose priori hypothesis to be tested.
Timing	The data are collected annually for the period of 1990-2011.
Data exclusions	No data are excluded from our analysis - we include all the data from the countries that have records in all the datasets.

Non-participation	There are no participants in this study and thus there is no dropouts/declined participants. The only reason that a country is not included in our analysis is because it does not have records in one or more datasets.
Randomization	n/a - this study does not conduct first-hand sampling and all the data for every country available in the dataset are used for the analysis to ensure the global representativeness. The data include global countries instead of using a randomized sampling strategy.

Reporting for specific materials, systems and methods

We require information from authors about some types of materials, experimental systems and methods used in many studies. Here, indicate whether each material, system or method listed is relevant to your study. If you are not sure if a list item applies to your research, read the appropriate section before selecting a response.

Materials & experimental systems

n/a	Involved in the study	
☒	☐	Antibodies
☒	☐	Eukaryotic cell lines
☒	☐	Palaeontology and archaeology
☒	☐	Animals and other organisms
☒	☐	Clinical data
☒	☐	Dual use research of concern

Methods

n/a	Involved in the study	
☒	☐	ChIP-seq
☒	☐	Flow cytometry
☒	☐	MRI-based neuroimaging
